# Validity of Patient-Reported Outcome Measures in Evaluating Nerve Damage Following Chemotherapy

**DOI:** 10.1001/jamanetworkopen.2024.24139

**Published:** 2024-08-09

**Authors:** Tiffany Li, Hannah C. Timmins, Fawaz M. Mahfouz, Terry Trinh, David Mizrahi, Lisa G. Horvath, Michelle Harrison, Peter Grimison, Michael Friedlander, Gavin Marx, Frances Boyle, David Wyld, Robert Henderson, Tracy King, Sally Baron-Hay, Matthew C. Kiernan, Claudia Rutherford, David Goldstein, Susanna B. Park

**Affiliations:** 1 School of Medical Sciences, Faculty of Medicine and Health, The University of Sydney, Sydney, Australia; 2 Neuroscience Research Australia, Sydney, Australia; 3 School of Clinical Medicine, University of New South Wales, Sydney, Australia; 4 The Daffodil Centre, The University of Sydney, a joint venture with Cancer Council New South Wales, Sydney, Australia; 5 Chris O’Brien Lifehouse, Sydney, Australia; 6 Sydney Medical School, University of Sydney, Sydney, Australia; 7 Prince of Wales Hospital, Sydney, Australia; 8 Sydney Adventist Hospital, Sydney, Australia; 9 School of Medicine and Psychology, The Australian National University, Canberra, Australia; 10 Patricia Ritchie Centre for Cancer Care and Research, Mater Hospital, Sydney, Australia; 11 Cancer Care Services, Royal Brisbane and Women’s Hospital, Faculty of Medicine, University of Queensland, Brisbane, Australia; 12 Faculty of Medicine, University of Queensland, Brisbane, Australia; 13 Department of Neurology, Royal Brisbane & Women’s Hospital, Brisbane, Australia; 14 Institute of Haematology, Royal Prince Alfred Hospital, Sydney, Australia; 15 Faculty of Medicine and Health, Cancer Care Research Unit, Susan Wakil School of Nursing and Midwifery, The University of Sydney, Sydney, Australia.; 16 Department of Medical Oncology, Royal North Shore Hospital, Sydney, Australia; 17 Brain and Mind Centre, University of Sydney, Australia; 18 Department of Neurology, Royal Prince Alfred Hospital, Sydney, Australia

## Abstract

**Importance:**

Chemotherapy-induced peripheral neuropathy (CIPN) is a substantial adverse effect of anticancer treatments. As such, the assessment of CIPN remains critically important in both research and clinic settings.

**Objective:**

To compare the validity of various patient-reported outcome measures (PROMs) with neurophysiological and sensory functional measures as the optimal method of CIPN assessment.

**Design, Setting, and Participants:**

This cohort study evaluated participants treated with neurotoxic chemotherapy across 2 cohorts using a dual-study design. Participants commencing treatment were assessed prospectively at beginning of neurotoxic treatment, midtreatment, and at the end of treatment. Participants who completed treatment up to 5 years prior were assessed cross-sectionally and completed a single assessment time point. Participants were recruited from oncology centers in Australia from August 2015 to November 2022. Data analysis occurred from February to November 2023.

**Exposures:**

Neurotoxic cancer treatment including taxanes, platinums, vinca-alkaloids, proteasome inhibitors, and thalidomide.

**Main Outcomes and Measures:**

CIPN was assessed via PROMs (European Organization for Research and Treatment of Cancer Quality of Life Questionnaire [EORTC-CIPN20], Functional Assessment of Cancer Therapy/Gynecological Cancer Group Neurotoxicity Questionnaire (FACT/GOG-Ntx), and the patient-reported outcomes version of the Common Terminology Criteria for Adverse Events [PRO-CTCAE]), neurological and neurophysiological assessment (Total Neuropathy Score and sural and tibial compound nerve amplitudes), and sensory measures (Grating orientation, Von Frey monofilament, and 2-point discrimination tasks). Core measurement properties of CIPN outcome measures were evaluated. Convergent and known-groups validity was assessed cross-sectionally following treatment completion, and responsiveness was evaluated prospectively during treatment. Neurological, neurophysiological, and sensory outcome measure scores were compared between those who reported high and low levels of CIPN symptoms using linear regressions.

**Results:**

A total of 1033 participants (median [IQR] age, 61 [50-59] years; 676 female [65.4%]) were recruited to this study, incorporating 1623 assessments. PROMs demonstrated best ability to accurately assess CIPN (convergent validity), especially the PRO-CTCAE composite score (*r* = 0.85; *P* < .001) and EORTC-CIPN20 (*r* = 0.79; *P* < .001). PROMS also demonstrated the best ability to discriminate between CIPN severity (known-groups validity) and to detect changes at onset of CIPN development (responsiveness), especially for EORTC-CIPN20 (*d* = 0.67; 95% CI, 0.52-0.83), FACT/GOG-Ntx (*d* = 0.65; 95% CI, 0.49-0.81) and the PRO-CTCAE (*d* = 0.83; 95% CI, 0.64-1.02). Other measures did not achieve threshold for convergent validity (α < 0.7). Neurophysiological and sensory measures did not demonstrate acceptable responsiveness. In regression models, neurological, neurophysiological, and sensory outcome measures were significantly impaired in participants who reported high levels of CIPN symptoms compared with those who reported low levels of CIPN symptoms.

**Conclusions and Relevance:**

In this cohort study of 1033 cancer patients, PROMs were the only measures to satisfy all 3 core measurement property criteria (convergent validity, known-groups validity, and responsiveness). These findings suggest that adoption of PROMs in clinical practice can equip clinicians with valuable information in assessing CIPN morbidity.

## Key Points

**Question:**

What is the optimal method of assessing chemotherapy-induced peripheral neuropathy (CIPN)?

**Findings:**

In this cohort study of 1033 participants with cancer, patient-reported outcome measures demonstrated superior ability to assess CIPN, discriminate between CIPN severity, and most sensitively capture development of CIPN symptoms over neurological and sensory functional approaches to CIPN assessment.

**Meaning:**

These findings suggest that patient-reported outcome measures are a valid and responsive method to assess CIPN, and adoption of these measures as the standardized assessment approach can optimize personalized medicine in clinical settings and improve translation of CIPN clinical trials.

## Teaser

This cohort study compares the validity of patient-reported outcome measures with neurophysiological and sensory functional measures for the assessment of chemotherapy-induced peripheral neuropathy among adults in Australia.

## Introduction

Peripheral nerve damage following cancer treatment is common, affecting up to 90% of patients exposed to neurotoxic chemotherapies including taxanes,^1,2^ platinums,^3^ vinca alkaloids,^4^ proteasome inhibitors^5^ and immunomodulatory drugs.^6^ Chemotherapy-induced peripheral neuropathy (CIPN) is a leading cause of dose reduction and premature treatment cessation, resulting in reduced treatment efficacy.^7,8^ Chronic symptoms may persist for years and are associated with long-term disability^9,10^ and increased fall risk.^11^

Despite this burden, it is becoming increasingly evident that the method of CIPN assessment represents a substantial barrier to improving outcomes in cancer survivors in both clinical practice and trials.^12^ In routine practice, clinicians need to be confident that alterations to treatment are made on the basis of an accurate representation of CIPN. Furthermore, there are an unparalleled number of potentially effective interventions ready for implementation in CIPN clinical trials^13,14,15,16^; however, a lack of successful trial methods and outcome measures have limited utility and translation. Until we can accurately measure CIPN, it will not be possible to demonstrate clinical benefit and understand symptom burden or extent of disability.

The National Cancer Institute Common Terminology Criteria for Adverse Events (NCI-CTCAE) peripheral neuropathy subscale is currently the most utilized measure of CIPN in both research and clinical practice settings.^17^ However, this measure is severely limited in the context of CIPN, including its lack of sensitivity to change and underreporting of symptom severity.^18,19^ In clinical settings, this presents a substantial issue because dose modification decisions are made on the basis of NCI-CTCAE grades. It is therefore crucial that a measure that can more accurately capture CIPN onset and severity and its impact be adopted as the standardized assessment tool.

There are currently more than 100 distinct CIPN measures,^20^ including patient-reported outcome measures (PROMs), clinical or neurological assessment, and functional or sensory assessment measures. Discordance between outcome measures have been previously highlighted,^21,22,23^ further complicating the selection of assessment tool.

PROMs are increasingly recognized as a valuable tool in cancer care broadly,^24,25^ as well as in toxic effects care and management.^26^ Their use in clinical research studies is growing, including in natural history studies^27,28^ and clinical trials.^29,30^ However, it is still unclear how PROMs compare with other types of CIPN assessments, and which measure should be considered the optimal CIPN assessment tool.

The present study aimed to comprehensively evaluate and compare core measurement properties of outcome measures developed to assess CIPN and establish validated standards for assessment. We sought to determine the optimal approach to assess CIPN and validate the role of PROMs as critical elements defining patient-relevant assessment of toxic effects of cancer therapy.

## Methods

### Participants and Study Design

This cohort study was approved by the Sydney Local Health District and South-Eastern Sydney Local Health District Human Research Ethics Committees and followed the Strengthening the Reporting of Observational Studies in Epidemiology (STROBE) reporting guideline. All participants provided informed signed consent in accordance with the Declaration of Helsinki.^31^ Participants were recruited into the study by their clinical care team from oncology centers in Australia from August 2015 to November 2022. Participants were eligible for analysis if they were adults and received 2 or more doses of neurotoxic cancer treatment (eg, taxanes, platinums, vinca-alkaloids, or proteasome inhibitors). The patient recruitment flowchart is provided in eFigure 1 in Supplement 1 and further detailed methods are provided in the eMethods in Supplement 1.

### Clinical Assessment

Participants commencing treatment were assessed prospectively at beginning of neurotoxic treatment, midtreatment, and at the end of treatment. Participants who completed treatment up to 5 years prior were assessed cross-sectionally and completed a single assessment time point. Participants completed a comprehensive battery of CIPN assessments as described later (further detailed in the eMethods in Supplement 1).

#### PROMs

The European Organization for Research and Treatment of Cancer Quality of Life CIPN Questionnaire (EORTC-CIPN20)^32^ has a transformed score range of 0 to 100, with higher scores indicating worse CIPN. The Functional Assessment of Cancer Therapy/Gynecological Cancer Group Neurotoxicity Questionnaire (FACT/GOG-Ntx)^33^ has a total score range of 0 to 52, where lower scores indicate worse CIPN.

The 2-item NCI-CTCAE-Numbness and Tingling questionnaire (ie, the patient-reported outcomes version of the CTCAE [PRO-CTCAE])^34^ investigating the severity and interference of CIPN was also evaluated (each item ranges from 0-4). A single numerical composite grade (range, 0-3) combining the 2 items was also calculated according to the developed algorithm.^35^

#### Clinical and Neurological Grading Scales

CIPN severity was graded by trained researchers using the NCI-CTCAE version 3 sensory neuropathy subscale (eFigure 2 in Supplement 1), on a scale from 0 to 4 (no symptoms to disabling). Training of assessors to grade this scale has been demonstrated to increase the tool’s accuracy and reproducibility,^36^ mitigating some limitations typically associated with the NCI-CTCAE.^18,19^ To reflect this, the researcher-graded NCI-CTCAE scale was termed RG-CTCAE for this analysis to draw distinction from the original scale as routinely collected in clinical practice.

Neurological assessment of nerve function was completed in upper and lower-limbs and graded by trained researchers using the Total Neuropathy Score, clinical version (TNSc; Johns Hopkins University).^37,38^ The total score ranged from 0 to 24, with higher scores indicating worse neuropathy.

#### Neurophysiological Assessment

As in previous studies,^27^ neurophysiological assessment of CIPN was completed by trained researchers with nerve conduction studies (NCS). Sural sensory nerve action potentials and tibial nerve compound muscle action potentials were recorded using standardized techniques.^39,40^

#### Sensory Measures

Measures of sensation elicited by trained researchers (T.L., H.C.T., F.M.M., T.T., D.M. and S.B.P.) including the Grating Orientation Task using JVP Domes (Stoelting Co)^41^ and the Von Frey monofilament task (Optihair2-Set; Marstock Nervtest)^42^ examined sensation at distal upper-limbs. The 2-point discriminator task^43^ assessed spatial sensation at distal lower-limbs.

### Statistical Analysis

Statistical analyses were performed from February to November 2023 using Stata version 14 (StataCorp). Descriptive findings are reported using mean (SD) or median (IQR) for nonparametric data determined using the Shapiro-Wilk test for normality. Comparisons between participant cohort data were calculated using *t* tests or Mann-Whitney U tests for nonparametric data and presented as mean (SE) or median (IQR). Significance was achieved at *P* < .05.

To identify optimal outcome measures, core measurement properties were evaluated^44,45^ and compared between the different outcome measures. Core measurement properties included ability to accurately assess its intended concept (ie, CIPN [convergent validity]), to discriminate between clinically distinct groups (known-groups validity), and to detect change in symptom development over time (responsiveness).

Convergent validity was defined as the extent to which an outcome measure was associated with other measures assessing the same construct. In the absence of an agreed optimal CIPN assessment tool, the comparator tool chosen to assess convergent validity was the RG-CTCAE for its discrete categorization of neuropathy severity and improved measurement properties over the NCI-CTCAE.^36^ Convergent validity was assessed by correlating outcome measures to the RG-CTCAE, with the acceptable threshold of Spearman correlation being greater than 0.7.

Known-groups validity referred to the extent to which outcome measures can differentiate between 2 clinically distinct groups. Clinically distinct groups in this study were defined as participants with no to mild CIPN (RG-CTCAE grade 0-1) vs moderate to severe CIPN (RG-CTCAE grade ≥2). Known-groups validity compared scores of each measure between clinically distinct groups using *t* tests.

Responsiveness for outcome measures was investigated in prospectively recruited participants assessed at baseline and midtreatment using Cohen *d* effect sizes (small = 0.2; moderate = 0.5; and high = 0.8) by calculating change in scores divided by pooled SDs. Baseline and midtreatment time points were chosen in order to evaluate which measures were sensitive to early changes at the onset of CIPN development.

In order to investigate whether high-CIPN symptom reporters have worse outcomes on quantitative measures of sensory dysfunction (NCS, TNSc, and sensory measures) compared with low-CIPN symptom reports, participants were categorized into tertiles according to reported severity on the EORTC-CIPN20. Analysis of outcome measures scores between the highest and lowest tertile of CIPN symptom reporters were completed using linear regressions controlling for age.

## Results

### Clinical Characteristics of Study Sample

A total of 1033 participants (median [IQR age, 61 [50-59] years; 676 female [65.4%]) were recruited to the study and completed 1623 assessment time points (eFigure 1 in Supplement 1). Participants predominantly had a diagnosis of breast cancer (320 participants [31.0%]) or colorectal and gastrointestinal cancers (230 participants [22.3%]) and received taxane (370 participants [35.8%]) or platinum (307 participants [29.7%]) chemotherapies (Table 1 and eTable 1 in Supplement 1). By midtreatment, 211 of the 335 prospectively recruited participants (63.0%) presented with CIPN (RG-CTCAE grade >0), with 46 (13.7%) having moderate-severe symptoms (RG-CTCAE grade ≥2). Overall CIPN data are provided in eTable 2 in Supplement 1. Further descriptive results are detailed in the eResults in Supplement 1.

**Table 1.** Participant Demographic and Clinical Characteristics for Total Cohort^a^

Characteristic	Participants, No. (%) (N = 1033)
Age, median (IQR), y	61 (50-69)
Sex	
Female	676 (65.4)
Male	357 (34.6)
Cancer type	
Breast	320 (31.0)
Colorectal and gastrointestinal	230 (22.3)
Gynecologic	204 (19.7)
Hematologic	113 (10.9)
Pancreatic	44 (4.3)
Prostate	42 (4.1)
Testicular	31 (3.0)
Head and neck	20 (1.9)
Other^b^	29 (2.8)
Cancer stage	
0-I	115 (11.1)
II	237 (33.0)
II	304 (29.4)
IV	226 (21.9)
Undefined^c^	151 (14.6)
Neurotoxic treatment	
Taxane	370 (35.8)
Platinum	307 (29.7)
Taxane or platinum	237 (22.9)
Vinca-alkaloids	56 (5.4)
Bortezomib	50 (4.8)
Thalidomide	7 (0.7)
Platinum or vinca-alkaloids	6 (0.6)

^a^ Demographic information for the total cohort, including prospective and cross-sectional recruitment groups, is outlined in eFigure 1 in Supplement 1.

^b^ Other included lung, bone, esophageal, brain, bladder, liver, or eye.

^c^ Nonsolid tumor or stage unavailable.

### Optimal Capture of CIPN: Convergent Validity

**Figure 1.**
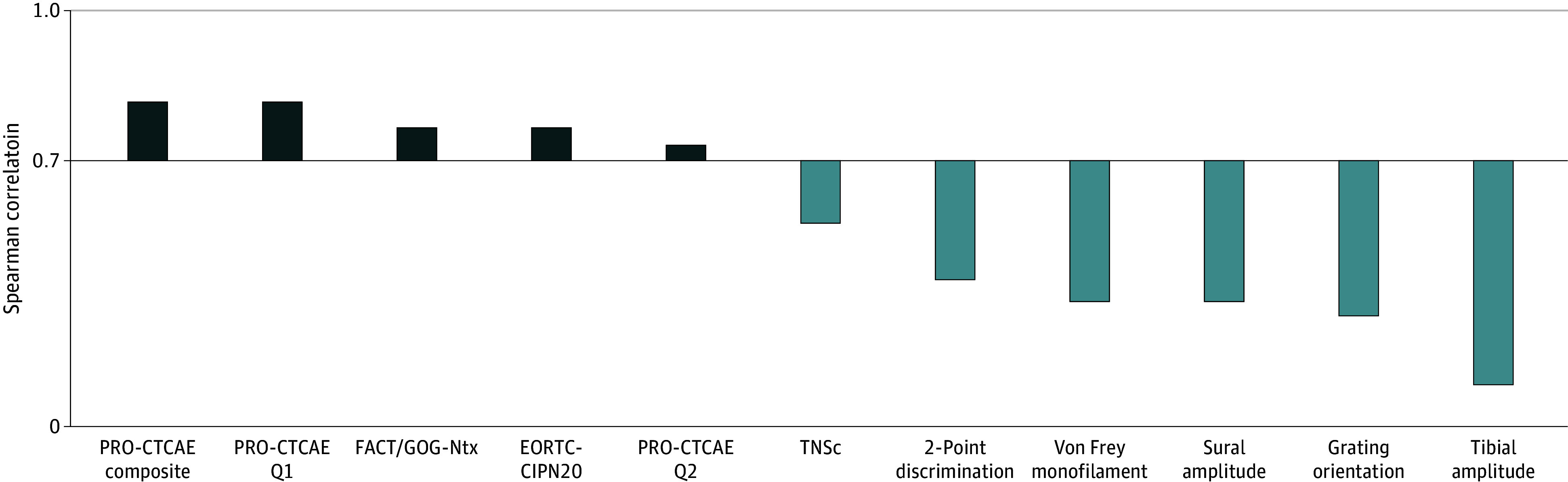
Spearman Correlation Values of Outcome Measures With RG-CTCAE Measures with correlations greater than 0.7 (above the x-axis) achieved the acceptable threshold for convergence validity. EORTC-CIPN20 indicates The European Organization for Research and Treatment of Cancer Quality of Life Chemotherapy-Induced Peripheral Neuropathy Questionnaire; FACT/GOG-Ntx, Functional Assessment of Cancer Therapy/Gynecological Cancer Group Neurotoxicity Questionnaire; PRO-CTCAE, National Cancer Institute Patient-Reported Outcomes Version of the Common Terminology Criteria for Adverse Events; Q, question; TNSc, Total Neuropathy Score, clinical version.

Convergent validity was evaluated by comparison of outcome measures with RG-CTCAE score (Figure 1). PROMs were all highly and significantly correlated with RG-CTCAE score demonstrating acceptable convergent validity. In particular, the correlations for PRO-CTCAE composite score (*r* = 0.85; *P* < .001) and EORTC-CIPN20 (*r* = 0.79; *P* < .001) were notably high. Other outcome measures did not meet the threshold for convergent validity. Neurological grading was only moderately correlated with RG-CTCAE (*r* = 0.57; *P* < .001), whereas neurophysiological and sensory functional measures demonstrated low to moderate correlation.

### Optimal Discrimination of CIPN Severity: Known-Groups Validity

Of the 953 participants assessed at posttreatment completion, 720 (75.6%) had CIPN, including 568 (59.6%) with no to mild CIPN and 385 (40.4%) with moderate to severe CIPN. A total of 293 participants (30.7%) had treatment dose modification due to CIPN. Mean scores for CIPN PROMs as well as other outcome measures were significantly worse for participants with moderate to severe compared with no to mild CIPN suggesting all measures demonstrated acceptable known-groups validity (Table 2).

**Table 2.** Known-Groups Validity Assessed in Cross-Sectional Study Group (N = 953)^a^

Outcome measure	Scores by CIPN symptom level, mean (SEM)		*P* value	Scores by dose modification, mean (SEM)		*P* value
	None-mild (n = 568)	Moderate-severe (n = 385)		No dose modification (n = 660)	Dose modification (n = 293)	
Neurological grading scale: TNSc	3.22 (0.10)	6.21 (0.16)	<.001	4.12 (0.12)	5.10 (0.18)	<.001
Neurophysiological assessment						
Sural nerve amplitude, μV	12.04 (0.39)	6.96 (0.34)	<.001	10.48 (0.34)	8.80 (0.47)	.003
Tibial nerve amplitude, mV	10.84 (0.23)	9.19 (0.26)	<.001	10.27 (0.21)	9.95 (0.33)	.20
Patient-reported outcome measures						
EORTC-CIPN20	7.56 (0.32)	25.11 (0.72)	<.001	13.04 (0.52)	18.25 (0.82)	<.001
FACT/GOG-Ntx	46.56 (0.22)	35.04 (0.44)	<.001	43.02 (0.34)	39.70 (0.52)	<.001
PRO-CTCAE Q1	0.73 (0.03)	2.24 (0.04)	<.001	1.20 (0.05)	1.63 (0.06)	<.001
PRO-CTCAE Q2	0.08 (0.01)	1.48 (0.06)	<.001	0.51 (0.04)	0.90 (0.07)	<.001
PRO-CTCAE composite	0.57 (0.02)	1.63 (0.04)	<.001	0.88 (0.03)	1.25 (0.05)	<.001
Sensory and functional measures						
Grating orientation task, mm	3.57 (0.07)	4.89 (0.14)	<.001	3.93 (0.08)	4.42 (0.15)	.001
Von Frey monofilament task, mN	0.42 (0.03)	4.43 (1.61)	.001	1.92 (0.87)	2.01 (0.48)	.48
2-Point discrimination task, mm	10.63 (0.16)	13.53 (0.18)	<.001	11.46 (0.16)	12.55 (0.21)	<.001

Abbreviations: CIPN, Chemotherapy-Induced Peripheral Neuropathy; EORTC-CIPN20, The European Organization for Research and Treatment of Cancer Quality of Life CIPN Questionnaire; FACT/GOG-Ntx, Functional Assessment of Cancer Therapy/Gynecological Cancer Group Neurotoxicity Questionnaire; PRO-CTCAE, National Cancer Institute Patient-Reported Outcomes Version of the Common Terminology Criteria for Adverse Events; Q, question; SEM, standard error of mean; TNSc, Total Neuropathy Score, clinical version.

^a^ Mean outcome measure scores by no to mild vs moderate to severe CIPN and whether participant had dose modification due to CIPN.

Dose modification due to CIPN was used as a surrogate for measuring significant CIPN during treatment to investigate neuropathy outcomes between participants who did receive dose modification due to neuropathy (293 participants [30.7%]) and those who did not (660 participants [69.3%]) (Table 2). All outcome measures reflected significantly worse CIPN outcomes in participants who received dose modification, except for tibial nerve amplitude and Von Frey monofilament (Table 2).

### Optimal Capture of Change Over Time: Responsiveness

**Figure 2.**
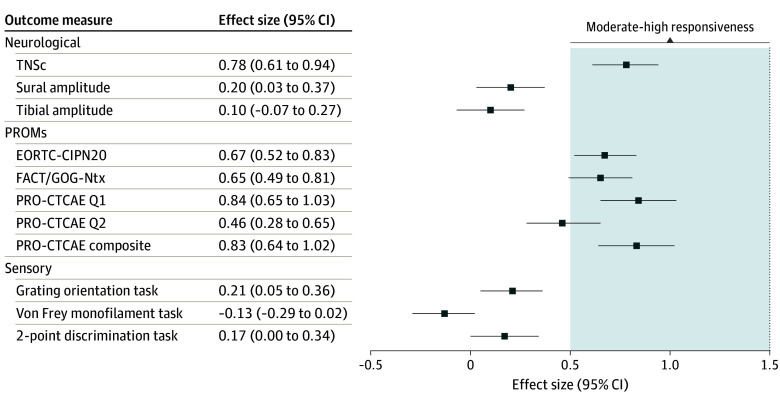
Responsiveness Effect Sizes for Outcome Measures Error bars denote 95% CIs while shaded area denotes moderate to high responsiveness, with moderate and high effect sizes defined as 0.5 and 0.8. EORTC-CIPN20 indicates The European Organization for Research and Treatment of Cancer Quality of Life Chemotherapy-Induced Peripheral Neuropathy Questionnaire; FACT/GOG-Ntx, Functional Assessment of Cancer Therapy/Gynecological Cancer Group Neurotoxicity Questionnaire; PRO-CTCAE, National Cancer Institute Patient-Reported Outcomes Version of the Common Terminology Criteria for Adverse Events; Q, question; TNSc, Total Neuropathy Score, clinical version.

Responsiveness was evaluated in 335 participants who completed baseline and midtreatment assessments (Figure 2). PROM scores demonstrated moderate to high effect sizes (EORTC-CIPN20, *d* = 0.67; 95% CI, 0.52-0.83; FACT.GOG-Ntx, *d* = 0.65; 95% CI, 0.49-0.81; PRO-CTCAE, *d* = 0.83; 95% CI, 0.64-1.02) indicating ability to detect change in CIPN symptom development. The RG-CTCAE scale also demonstrated high responsiveness (*d* = 0.91; 95% CI, 0.75-1.10). TNSc demonstrated a moderate effect size (*d* = 0.78; 95% CI, 0.61-0.94); however, secondary analysis identified that the 2 patient-reported symptom items of the TNSc may have skewed results, with the effect size dropping (*d* = 0.52; 95% CI, 0.36-0.68) when the patient-reported items were omitted and only neuropathic signs (pinprick, vibration, strength, and reflexes) were included. Neurophysiological and sensory functional assessments did not demonstrate acceptable responsiveness.

### Analysis of Patients With Low-Level vs High-Level CIPN Symptoms 

As a further means of demonstrating validity of PROMs for CIPN assessment, we investigated if participants who reported high levels of CIPN symptoms displayed different degrees of sensory nerve dysfunction on the NCS, TNSc, and sensory function tests compared with participants who reported low levels of CIPN symptoms. To distinguish between those with high-level and low-level CIPN symptoms, participants were categorized into tertiles according to reported severity on the EORTC-CIPN20 (low-tertile, 2.3 ± 2.0; mid-tertile, 11.0 ± 2.9; high-tertile, 29.4 ± 12.7).

**Figure 3.**
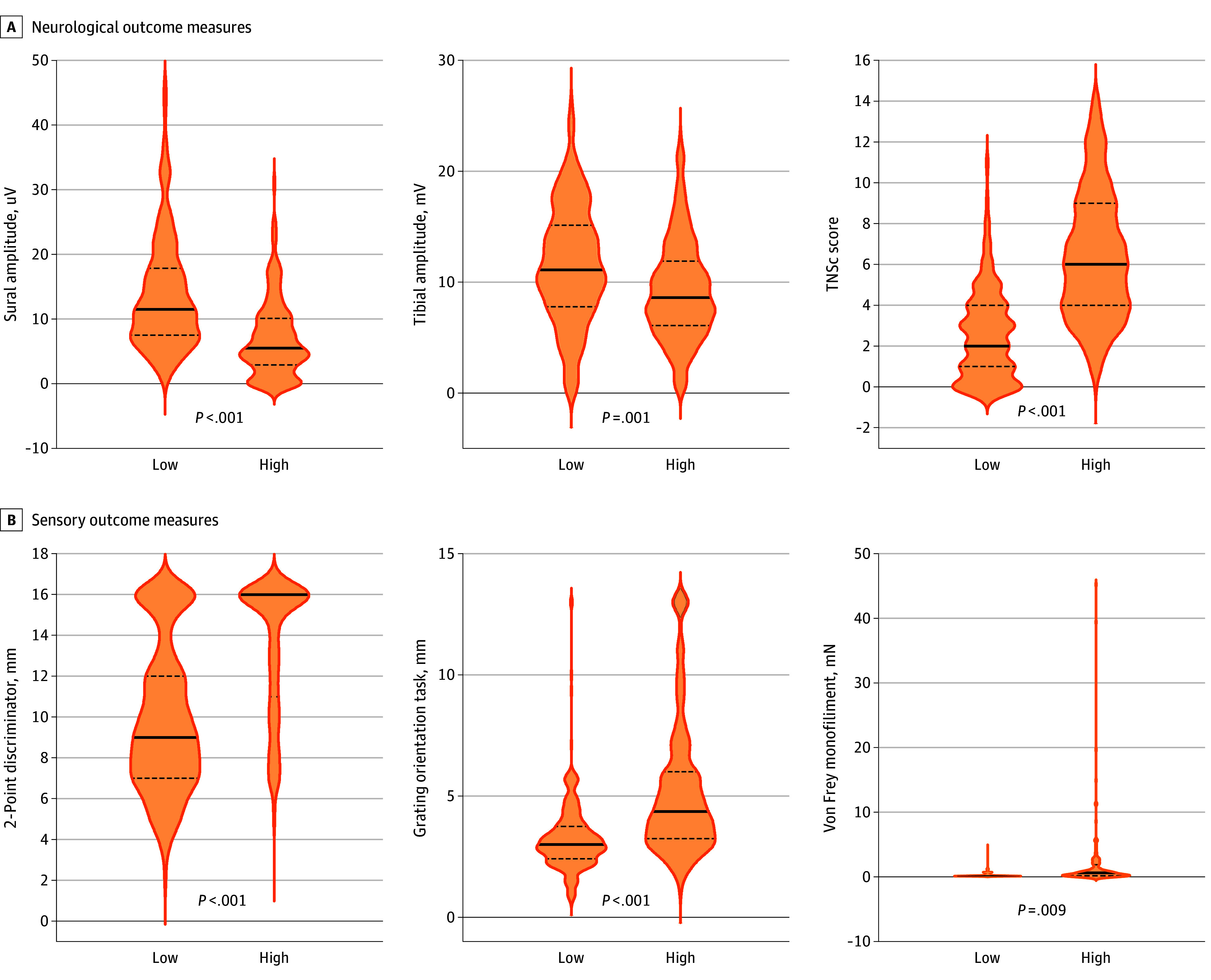
Violin Plot of Chemotherapy-Induced Peripheral Neuropathy Outcome Measure Scores Between Low vs High Symptom Reporters The thick dotted lines indicates the median score and the thin dotted lines indicates the 25th and 75th percentile. TNSc indicates Total Neuropathy Score, clinical version.

Neurophysiological (sural and tibial amplitudes), neurological (TNSc), and functional (Grating Orientation Task, 2-point discrimination task, and Von Frey monofilament task) CIPN outcome measures were significantly worse for those who reported high levels of CIPN symptoms compared with those who reported low levels (Figure 3), and this finding remained significant after controlling for age (eTable 3 in Supplement 1). This finding suggests that participants reporting more severe symptoms also demonstrated worse objective, sensory, and neurophysiological CIPN outcomes.

## Discussion

The present series of analyses in this cohort study comprehensively compared and evaluated patient-reported, neurophysiological, and sensory and functional CIPN outcome measures. PROMs were the only measures to satisfy all 3 core measurement property criteria: capability to accurately assess CIPN (convergent validity), distinguish between CIPN severity (known-groups validity), and sensitively capture development of CIPN symptoms (eFigure 3 in Supplement 1).

### Impact of Neurotoxic Cancer Treatment

The majority of participants (63.0%) had developed CIPN by midtreatment in this study, underscoring its high prevalence as a cancer treatment toxic effect. More than 30% of participants received a dose reduction or treatment discontinuation due to CIPN, highlighting the clinical impact of CIPN during treatment. In line with previous studies,^46,47^ 75.6% of participants reported persistent toxic effects following treatment, with moderate to severe symptoms in 40.4%, indicating the need for long-term management pathways.^48^

### Neuropathy Assessment in Clinical Practice

Accurate assessment of CIPN is important in clinical practice, and this need is amplified when considering its repercussions on dose modification.^49^ Recent studies^50,51,52^ have suggested equivocal treatment efficacy with reduced neurotoxic treatment exposure, leading to the issue of when to cease treatment. As the landscape for treatments continues to evolve, it is essential to equip oncologists with optimal tools to make informed decisions.

In current practice, dose modification decisions are typically based on the patient’s NCI-CTCAE grade, which is informed by nonstandardized patient-clinician symptom communication. There are numerous pitfalls associated with NCI-CTCAE grading, including disagreement between raters,^53^ underestimation of neuropathy severity,^54^ and low sensitivity to change.^54^ We propose a revision to how CIPN is assessed in clinical practice. Incorporation of validated PROMs would provide a standardized benchmark for CIPN assessment, providing clinicians with an improved framework for decision making. In line with this proposal, early evidence^55^ has suggested that personalized dose adjustment associated with PROM data may reduce severity of CIPN. Future studies should investigate how cutoff scores in CIPN PROMs can be used to inform and predict dose modification decisions.

This study highlighted the benefits and strengths of harnessing patient report in CIPN assessment and management. Patient report has a critically important role in cancer care more broadly,^56^ and adoption of PROMs to clinical practice is supported by international consensus guidelines.^57^ Use of PROMs in cancer care is associated with significant benefits, including less emergency admittance and hospitalization, and longer duration of chemotherapy.^25^

Prior studies have validated PROMs for CIPN assessment^26^ and defined acceptable psychometric properties, including validity and responsiveness,^33,58,59,60^ but have not determined how their psychometric properties may make them more suitable for CIPN assessment. Our large-scale study identified that CIPN PROMs were suitable to detect change and accurately able to measure CIPN, in comparison with sensory, neurological examination, and neurophysiological outcomes. Accordingly, these findings support the recommendation to adopt these tools into routine clinical practice. While there may be insufficient time to administer 13- or 20- item PROMs such as the FACT/GOG-Ntx and EORTC-CIPN20 routinely, the shorter 2-item PRO-CTCAE^61^ offers an alternative, time-efficient assessment. This measure also demonstrated strong measurement properties in the current study and has previously been recommended for use in clinical practice to promote shared decision-making between clinicians and patients.^62^

### Considerations on the Use of PROMs

The assessment of neuropathy using PROMs require personalized assessment of symptom severity and burden, which differs between individuals, as well as from clinicians who have a broader perspective of the range of symptom severity.^63^ However, the present study has validated the patient’s perspective by demonstrating that objective measures reflected worse neuropathy in tandem with higher reported CIPN symptoms.

Another important consideration is the lack of predefined cutoff scores for mild, moderate, or severe neuropathy. While scores associated with clinically important change have been determined for CIPN PROMs,^64^ further work is required to guide interpretation in the clinic. In addition, gaps have been identified in the coverage of CIPN PROMs,^26^ which may limit the ability of existing tools to identify disability associated with CIPN, including sleep disturbance and balance impairment.

Successful integration of PROMs may be limited by language barriers and willingness of engagement by patients and clinicians. Successful embedding of CIPN PROMs into routine clinical practice requires addressing systemic barriers including personnel availability, time constraints, and integration into clinical workflows.^65,66^ To facilitate use of PROMS, the development of robust systems to collect PROMs electronically and automatically report findings to clinicians upon completion are necessary.^48,67^

### Neuropathy Assessment in Research Settings

Selection of the most appropriate CIPN outcome measure will depend on the setting. Assessing CIPN in clinical research requires different considerations than those in clinical care. Furthermore, the requirements of a neuropathy measure to comprehensively phenotype CIPN in a natural history study will differ from a clinical trial where CIPN is one of many adverse events being monitored. Clinical research studies or trials aiming to improve understanding and prevent or reverse axonal damage relating to CIPN may need to investigate physiological biomarkers as outcome measures. Neurofilament light chain, a blood-based biomarker of axonal damage, has been demonstrated to be elevated in patients with CIPN^68^ and suggests that this elevation may precede clinical symptom development.^69^

The TNS has previously been suggested to be a valid and responsive method of assessing CIPN.^60,70^ However in the present study, the TNSc did not reach threshold for convergent validity, and responsiveness deteriorated once the patient-reported symptom items were omitted (effect size dropping from 0.8 to 0.5), suggesting neuropathic sign-components alone are less optimal at detecting CIPN development. Similarly, sensory assessments did not strongly correlate with RG-CTCAE grade and did not respond to change in CIPN symptoms over time. Sensory assessments evaluated neuropathy at specific focal locations, such as the thumb or toe, rather than providing a global assessment of symptom severity, which may reduce responsiveness. In addition, it is important to recognize that varying approaches to CIPN assessment may be examining different constructs. Where PROMs are investigating overall severity and impact of CIPN, neurological and sensory assessments are often examining discrete domains, which are less likely to have convergent validity with global neuropathy status.

Similarly, NCS are a focal diagnostic tool for identifying peripheral neuropathies including CIPN.^71,72^ Results from this longitudinal analysis suggested that NCS are not sensitive to capture early signs of degeneration because reduction in sensory amplitudes may occur after development of neuropathy symptoms.^73^ In the present study, NCS were completed at discrete sites of the lower-limb, which may limit their ability to be indicative of overall neuropathy status. In addition, due to the large range of normative values,^74^ NCS at a single time point may also not be sensitive to identify abnormal results. Future studies should investigate whether a battery of upper- and lower-limb NCS measures is better able to provide a more accurate neurophysiologic picture of CIPN morbidity. While NCS is not recommended as a measure of diagnosing or detecting early development of CIPN in routine clinical practice, it may provide utility in the examination of axonal degeneration in CIPN research studies.

### Limitations

This study has limitations. In the present study, clinical grading of CIPN was completed by researchers. Although that is not how the NCI-CTCAE is traditionally used in clinical practice, the training in neuropathy grading among researchers mitigated limitations typically associated with the NCI-CTCAE.^36^ This consistency in grading likely led to the RG-CTCAE demonstrating uncharacteristically high responsiveness. This study included a large, heterogenous clinical population, with multiple cancer and chemotherapy types. While this heterogeneity was deliberate to include the breadth of patients treated with neurotoxic agents in routine practice, specific findings related to individual tumor or chemotherapy groups were not evaluated.

## Conclusions

This cohort study found that clinically informative measurement properties of PROMs were superior to assess neurotoxic effects over other assessment methods. Accordingly, the adoption of CIPN PROMs is recommended in clinical practice, as well as future clinical trial initiatives. The incorporation of patient perspective will enhance therapeutic decision-making and promote precision medicine approaches, leading to improved long-term neuropathy outcomes for cancer patients.

## Notes

Supplement 1. **eFigure 1.** Patient Recruitment Flowchart
eMethods.
**eFigure 2.** National Cancer Institute-Common Terminology Criteria for Adverse Events (NCI-CTCAE) Version 3, Sensory Neuropathy Subscale **eTable 1**. Breakdown of Neurotoxic Chemotherapy Treatments, Total N=1033 **eTable 2.** Overall CIPN Outcomes in Patients Assessed Post Treatment Completion (N=953)
eResults.
**eTable 3.** Regression Analyses of CIPN Outcomes by Low-, Mid- or High-CIPN Responders, Controlling for Age **eFigure 3.** Summary of Results

Supplement 2.
Data Sharing Statement
