# Supplementary material for: Validity of Patient-Reported Outcome Measures in Evaluating Nerve Damage Following Chemotherapy
Source: JAMA Netw Open. 2024 Aug 9;7(8):e2424139. doi: 10.1001/jamanetworkopen.2024.24139 (PMC11316238; doi:10.1001/jamanetworkopen.2024.24139)
Supplement: Supplement 1. — eFigure 1. Patient Recruitment Flowchart eMethods. eFigure 2. National Cancer Institute-Common Terminology Criteria for Adverse Events (NCI-CTCAE) Version 3, Sensory Neuropathy Subscale eTable 1. Breakdown of Neurotoxic Chemotherapy Treatments, Total N=1033 eTable 2. Overall CIPN Outcomes in Patients Assessed Post Treatment Completion (N=953) eResults. eTable 3. Regression Analyses of CIPN Outcomes by Low-, Mid- or High-CIPN Responders, Controlling for Age eFigure 3. Summary of Results [file jamanetwopen-e2424139-s001.pdf]

Supplemental Online Content

Li T, Timmins HC, Mahfouz FM, et al. Validity of patient-reported outcome measures in evaluating nerve damage following chemotherapy. *JAMA Netw. Open*. 2024;7(8):e2424139. doi:10.1001/jamanetworkopen.2024.24139

eFigure 1. Patient Recruitment Flowchart

eMethods

eFigure 2. National Cancer Institute-Common Terminology Criteria for Adverse Events (NCI-CTCAE) Version 3, Sensory Neuropathy Subscale

eTable 1. Breakdown of Neurotoxic Chemotherapy Treatments, Total N=1033

eTable 2. Overall CIPN Outcomes in Patients Assessed Post Treatment Completion (N=953)

eResults

eTable 3. Regression Analyses of CIPN Outcomes by Low-, Mid- or High-CIPN Responders, Controlling for Age

eFigure 3. Summary of Results

This supplemental material has been provided by the authors to give readers additional information about their work.

© 2024 Li T et al. *JAMA Network Open.*

eFigure 1. Patient Recruitment Flowchart

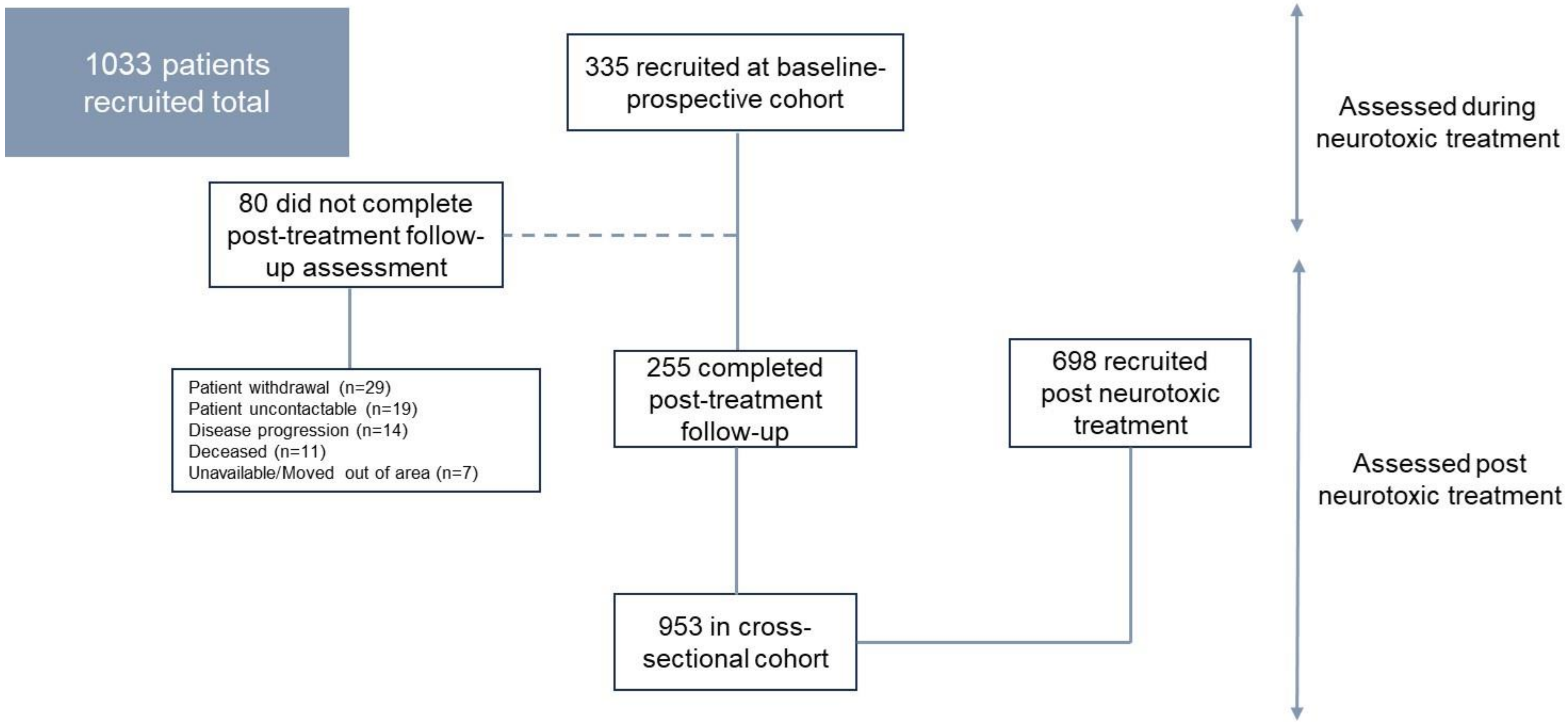

© 2024 Li T et al. *JAMA Network Open.*

eFigure 2. National Cancer Institute-Common Terminology Criteria for Adverse Events (NCI-CTCAE) Version 3, Sensory Neuropathy Subscale

**Grade 0**	**Grade 1**	**Grade 2**	**Grade 3**	**Grade 4**
No Symptoms	Asymptomatic; loss of deep tendon reflexes or paresthesia (including tingling) but not interfering with function	Sensory alteration or paresthesia (including tingling), interfering with function, but not interfering with ADL	Sensory alteration or paresthesia interfering with ADL	Disabling

© 2024 Li T et al. *JAMA Network Open.*

eFigure 3. Summary of Results

Matrix indicating which outcome measures achieved threshold for convergent validity, known-groups validity and responsiveness

	Convergent Validity	Known-Groups Validity	Responsiveness
**Neurological Grading Scale**			
TNSc		YES	YES
**Neurophysiological Assessment**			
Sural nerve amplitude		YES	
Tibial nerve amplitude		YES	
**PROMs**			
EORTC-CIPN20	YES	YES	YES
FACT/GOG-Ntx	YES	YES	YES
PRO CTCAE q1	YES	YES	YES
PRO CTCAE q2	YES	YES	YES
PRO CTCAE composite	YES	YES	YES
**Sensory and Functional Measures**			
Grating orientation task		YES	
Von Frey monofilament task		YES	
2-point discrimination task		YES	

© 2024 Li T et al. *JAMA Network Open.*

eMethods

The prospective study arm recruited participants at commencement of neurotoxic treatment. Participants were assessed longitudinally with study visits prior to first or second neurotoxic treatment dose (baseline), midway through neurotoxic treatment protocol (mid-treatment) and at-completion of final neurotoxic treatment (end-of-treatment). End-of-treatment timepoint was undertaken at the time of final neurotoxic treatment, and moved from intended timing if the final treatment was premature or delayed.

The cross-sectional study arm recruited participants following completion of neurotoxic treatment. Participants completed neurotoxic treatment up to five years prior, and were assessed at a single timepoint post-treatment completion.

The study was approved by the Sydney Local Health District and South-Eastern Sydney Local Health District Human Research Ethics Committees. All participants provided informed signed consent in accordance with the Declaration of Helsinki. This study followed the Strengthening the Reporting of Observational Studies in Epidemiology (STROBE) reporting guidelines[31].

Demographic and treatment dosing information were obtained from medical records. Dose modifications of neurotoxic agents (including reduction and early cessation) were identified and classified by whether they were due to CIPN.

CIPN Assessment Tools

Patient Reported Outcome Measures

The European Organization for Research and Treatment of Cancer Quality of Life Chemotherapy-Induced Peripheral Neuropathy Questionnaire (EORTC-CIPN20) is a widely used validated measure of CIPN[32]. This 20-item questionnaire assesses sensory, motor and autonomic neuropathy with each item scored on a 4-point scale (1-4, not at all – very much). Total scores are transformed to a 0-100 scale with greater scores indicating worse neuropathy.

Along with the EORTC-CIPN20, the Functional Assessment of Cancer Therapy/Gynecological Cancer Group Neurotoxicity questionnaire (FACT/GOG-Ntx) make up the two most commonly used, validated CIPN PROMs. The FACT/GOG-Ntx is a 13-item measure with each item scored on a 5-point scale (0=Not at all, 4=Very much)[33]. Items are reverse scored with a lower total score indicating greater CIPN (total score range 0-52).

The National Cancer Institute Patient-Reported Outcome Common Terminology Criteria for Adverse Events- Numbness & tingling (PRO-CTCAE) is a PROM designed to evaluate cancer treatment toxicities, complementing the NCI-CTCAE[34]. The PRO-CTCAE sensory neuropathy subscale consists of two items. The first assesses numbness and tingling in the hands and feet (0=None, 4=Very severe), and the second assessing the degree which these symptoms interfere with daily activities (0=Not at all, 4=Very much). A single numerical composite grade combining the two items was also calculated according to the developed algorithm[35].

Clinical and Neurological Grading Scales

Clinical CIPN was graded by trained researchers using the National Cancer Institute Common Terminology Criteria for Adverse Events peripheral neuropathy subscale (RG-

© 2024 Li T et al. *JAMA Network Open.*

CTCAE). As previously highlighted, grading by trained researchers increases the tool's accuracy and reproducibility[36], mitigating limitations typically associated with the NCI-CTCAE[18,19]. Version 3 of this tool was used, grading CIPN on a scale from 0 (no symptoms) to 4 (disabling).

Neurological assessment of nerve function was completed in upper- and lower-limbs using the Total Neuropathy Score, clinical version (TNSc, © Johns Hopkins University). The TNSc is a validated composite measure of CIPN[37,38], consisting of two patient reported items assessing the extent of sensory and motor symptoms as well as four items assessing quantifiable neuropathic signs (pinprick, vibration sensation, strength and deep tendon reflexes). Each item is scored 0-4 for a total score range of 0-24, with higher scores indicating greater neuropathy.

Neurophysiological Assessment

Objective assessment of CIPN was completed with nerve conduction studies (NCS) on the left side of the lower limbs using a Nicolet EDX Synergy device (Natus Medical, Inc., Pleasanton, California). NCS consisted of antidromic sural sensory nerve action potentials (SNAPs), recorded at the lateral malleolus with the stimulation site 10–15 cm proximal and tibial nerve compound muscle action potentials (CMAPs) recorded from the abductor hallucis muscle, stimulating posterior to the medial malleolus.

Sensory Measures

The Grating Orientation Task (GOT) was used to assess tactile sensation on the fingertips using JVP Domes (Stoelting Co, Illinois, USA), with dome gratings ranging between 0.75mm to 12mm wide. Domes were pressed onto the index finger of the dominant hand either proximal-distally or lateral-medially in random order to identify the smallest grating that could be reliably discriminated[41].

Mechanical detection threshold of fingertips was assessed using the Von Frey Monofilament task (VF) (Optihair2- Set, Marstock Nervtest, Germany). Filaments exerting forces ranging 0.125-512 mN were pressed on the index finder of the dominant hand and patients were told to acknowledge when the filament was felt. Five threshold determinations were made, each with a series of ascending and descending stimulus intensities. The final threshold was the geometric mean of these five series[42].

The 2-Point Discriminator Task (2PD) was used to assess spatial sensation in distal lower limbs. An aesthesiometer was placed on the first toe of the left foot and participants were required to correctly differentiate between 1 and 2 points (distance of the 2 points ranged 2-15 mm)[43]. The final score was the smallest 2-point distance that can be correctly discriminated 7 out of 10 trials.

© 2024 Li T et al. *JAMA Network Open.*

eResults

Descriptive Results

There were no differences in demographics between participants recruited following completion of neurotoxic treatment (n=698) and participants recruited at beginning of treatment (n=335, P>0.05), except for older age at time of assessment in cohort recruited after treatment completion (62 (52-70) vs 57 (47-66) years, P<0.05).

Participants assessed following completion of neurotoxic treatment (n=953) were included in convergent and known-groups validity analyses and had completed neurotoxic treatment 11.6±12.2 months prior to assessment. 30.7% (n=293) of participants had treatment dose modification due to CIPN. Overall CIPN data is presented in Supplementary Table 2. 75.6% (n=720) of participants had CIPN at time of assessment (Grade>0), with 40.4% (n=385) experiencing moderate to severe symptoms (Grade ≥2).

Prospectively recruited participants (n=335) were included in responsiveness analysis. These participants were assessed at baseline (either prior to first (n=157) or second neurotoxic (n=178 treatment dose) and at mid-treatment (8.9±4.7 weeks after baseline). Patient-reported symptoms were not significantly different at baseline between participants assessed prior to first and second neurotoxic infusion (EORTC-CIPN20, FACT/GOG-Ntx, PRO-CTCAE; P>0.05). By mid-treatment, 63.0% (n=211) presented with CIPN (Grade>0 RG-CTCAE), with 13.7% (n=46) having moderate-severe symptoms (Grade ≥2 RG-CTCAE).

© 2024 Li T et al. *JAMA Network Open.*

eTable 1. Breakdown of Neurotoxic Chemotherapy Treatments, Total N=1033

**Neurotoxic treatment**	**n (%)**
Oxaliplatin	237 (22.9%)
Paclitaxel	231 (22.4%)
Paclitaxel + Carboplatin	201 (19.4%)
Docetaxel	106 (10.3%)
Cisplatin	70 (6.8%)
Vinca-alkaloids	56 (5.4%)
Bortezomib	50 (4.8%)
Abraxane	24 (2.3%)
Docetaxel + Carboplatin	9 (0.9%)
Oxaliplatin + Abraxane	9 (0.9%)
Docetaxel + Oxaliplatin	9 (0.9%)
Thalidomide	7 (0.7%)
Paclitaxel + Cisplatin	6 (0.6%)
Cisplatin + Vinca-alkaloid	6 (0.6%)
Paclitaxel + Docetaxel	5 (0.5%)
Docetaxel + Cisplatin	2 (0.2%)
Docetaxel + Paclitaxel	2 (0.2%)
Paclitaxel + Abraxane	2 (0.2%)
Oxaliplatin + Paclitaxel	1 (0.1%)

© 2024 Li T et al. *JAMA Network Open.*

eTable 2. Overall CIPN Outcomes in Patients Assessed Post Treatment Completion (N=953)

**CIPN Outcome Measure**	**N (%)**
RG-CTCAE	
Grade 0	233 (24.4%)
Grade 1	335 (35.2%)
Grade 2	340 (35.7%)
Grade 3	43 (4.5%)
Grade 4	2 (0.2%)
	**Median (IQR)**
TNSc	4 (4)
Sural amplitude (μV)	8 (9)
Tibial amplitude (mV)	9.9 (6.9)
EORTC-CIPN20	11.1 (17.5)
FACT/GOG-Ntx	44 (12)
PRO-CTCAE Q1	1 (2)
PRO-CTCAE Q2	0 (1)
PRO-CTCAE composite	1 (1)
Grating Orientation Task (mm)	3.5 (2.0)
Von Frey Monofilament Task (mN)	0.2 (0.6)
2-Point Discrimination Task (mm)	12 (8)

© 2024 Li T et al. *JAMA Network Open.*

eTable 3. Regression Analyses of CIPN Outcomes by Low-, Mid- or High-CIPN Responders, Controlling for Age

CIPN Measure	Coefficient	Standard Error	P	95% Confidence Interval	
**Sural amplitude**					
High vs low responder	-2.78	0. 32	<0.001	-3.42	-2.15
Age	-0.17	0.02	<0.001	-0.21	-0.13
**Tibial amplitude**					
High vs low responder	-0.64	0.21	<0.005	-1.5	-0.24
Age	-0.15	0.02	<0.001	-0.18	-0.12
**TNSc**					
High vs low responder	1.72	0.11	<0.001	1.50	1.93
Age	0.06	0.01	<0.001	0.05	0.07
**2-Point Discriminator Task**					
High vs low responder	1.33	0.14	<0.001	1.06	1.60
Age	0.12	0.01	<0.001	0.10	0.14
**Grating Orientation Task**					
High vs low responder	0.79	0.0	<0.001	0.61	0.97
Age	0.04	0.01	<0.001	0.03	0.06
**Von Frey Monofilament Task**					
High vs low responder	2.08	1.01	<0.05	0.10	4.05
Age	0.06	0.08	0.41	-0.09	0.21

© 2024 Li T et al. *JAMA Network Open.*
