# Supplementary material for: Validity of Patient-Reported Outcome Measures in Evaluating Nerve Damage Following Chemotherapy
Source: JAMA Netw Open. 2024 Aug 9;7(8):e2424139. doi: 10.1001/jamanetworkopen.2024.24139 (PMC11316238; doi:10.1001/jamanetworkopen.2024.24139)
Supplement: Supplement 2. — Data Sharing Statement [file jamanetwopen-e2424139-s002.pdf]

Data Sharing Statement

Li. Validity of Patient-Reported Outcome Measures in Evaluating Nerve Damage Following Chemotherapy. *JAMA Netw Open*. Published August 09, 2024.
doi:10.1001/jamanetworkopen.2024.24139

Data
Data available: Yes
Data types: Deidentified participant data
How to access data: Data may be available upon request to susanna.park@sydney.edu.au
When available: With publication

Supporting Documents
Document types: None

Additional Information
Who can access the data: Researchers whose proposed use of the data has been approved
Types of analyses: For any research purpose
Mechanisms of data availability: With a signed data access agreement
